# Investigating the Spatio‐Temporal Signatures of Language Control–Related Brain Synchronization Processes

**DOI:** 10.1002/hbm.70109

**Published:** 2025-01-21

**Authors:** Alexandru Mihai Dumitrescu, Tim Coolen, Vincent Wens, Antonin Rovai, Nicola Trotta, Serge Goldman, Xavier De Tiège, Charline Urbain

**Affiliations:** ^1^ Université libre de Bruxelles (ULB), UNI – ULB Neuroscience Institute Laboratoire de Neuroanatomie et Neuroimagerie translationnelles (LN2T) Brussels Belgium; ^2^ Université libre de Bruxelles (ULB), Hôpital Universitaire de Bruxelles (H.U.B.), CUB Hôpital Erasme Department of Radiology Brussels Belgium; ^3^ Université libre de Bruxelles (ULB), Hôpital Universitaire de Bruxelles (H.U.B.), CUB Hôpital Erasme Service of Translational Neuroimaging Brussels Belgium; ^4^ Université libre de Bruxelles (ULB), Hôpital Universitaire de Bruxelles (H.U.B.), CUB Hôpital Erasme Department of Nuclear Medicine Brussels Belgium; ^5^ Université libre de Bruxelles (ULB), UNI – ULB Neuroscience Institute, Neuropsychology and Functional Neuroimaging Research Unit (UR2NF) Center for Research in Cognition and Neurosciences (CRCN) Brussels Belgium

**Keywords:** brain synchronization mechanisms, functional magnetic resonance imaging, language control processes, magnetoencephalography, spatio‐temporal brain dynamics

## Abstract

Language control processes allow for the flexible manipulation and access to context‐appropriate verbal representations. Functional magnetic resonance imaging (fMRI) studies have localized the brain regions involved in language control processes usually by comparing high vs. low lexical–semantic control conditions during verbal tasks. Yet, the spectro‐temporal dynamics of associated brain processes remain unexplored, preventing a proper understanding of the neural bases of language control mechanisms. To do so, we recorded functional brain activity using magnetoencephalography (MEG) and fMRI, while 30 healthy participants performed a silent verb generation (VGEN) and a picture naming (PN) task upon confrontation with pictures requiring low or high lexical–semantic control processes. fMRI confirmed the association between stronger language control processes and increased left inferior frontal gyrus (IFG) perfusion, while MEG revealed these controlled mechanisms to be associated with a specific sequence of early (< 500 ms) and late (> 500 ms) beta‐band (de)synchronization processes within fronto‐temporo‐parietal areas. Particularly, beta‐band modulations of event‐related (de)synchronization mechanisms were first observed in the right IFG, followed by bilateral IFG and temporo‐parietal brain regions. Altogether, these results suggest that beyond a specific recruitment of inferior frontal brain regions, language control mechanisms rely on a complex temporal sequence of beta‐band oscillatory mechanisms over antero‐posterior areas.

## Graphical abstract

The study aimed at investigating the oscillatory brain dynamics underlying language control processes, a mechanism that critically allows the flexible access to context‐relevant representations during language production tasks. We showed that language control processes rely on a complex sequence of beta‐band brain synchronization processes encompassing fronto‐temporo‐parietal brain regions, with the prefrontal areas being particularly involved at early stages (< 500 ms).

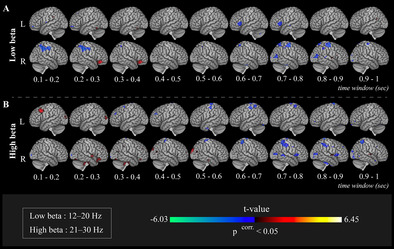

## 1 Introduction

Lexical–semantic control mechanisms are critical for optimal language production as they allow for the flexible selection and access to context‐appropriate representations (e.g., a *flag* can be *waved* or *planted*, depending on the situation) [Jackson 2021; Jefferies 2013]. The localization of these language control mechanisms has been mainly characterized using functional magnetic resonance imaging (fMRI) studies conducted in healthy adult participants or brain‐injured patients in the context of verb generation (VGEN) tasks. Participants were asked to silently generate a verb upon confrontation with a visually presented word or image that triggered high vs. low levels of competition between lexical–semantic representations. High control (HC) conditions involved high lexical–semantic control processes toward verb selection (e.g., the word *door* being associated with multiple potential verb responses: *open*, *close*, *slam*, *lock*, *slide*, *push*), while low control (LC) conditions relied on more automatic verb selection processes (e.g., the word *bed* being associated with a few possible verb responses: *sleep* or *relax*).

Globally, these fMRI studies reported a stronger involvement of the left inferior frontal gyrus (LIFG, often analyzed as a region of interest (ROI)) in HC compared with LC lexical–semantic conditions [Thompson‐Schill, D'Esposito, Aguirre, and Farah 1997; Thompson‐Schill et al. 1998; Thompson‐Schill, D'Esposito, and Kan 1999], suggesting a specific role of frontal brain regions in language control processes. This was further investigated in a subsequent fMRI study showing that a VGEN task triggered stronger activations of the left ventrolateral prefrontal cortex, the anterior cingulate cortex, and the insula compared with a picture naming (PN) task, the latter suggested to be less dependent on language control processes [Bourguignon et al. 2018]. Accordingly, while the VGEN task requires a controlled access and selection of context‐relevant representations among several possible lexical–semantic alternatives, the PN task relies more on a less‐controlled bottom‐up selection of words out of a restricted sets of competitors [Bourguignon et al. 2018].

Still, language control processes also rely on a more widespread network as, in addition to the IFG and the dorso‐medial prefrontal cortex, the posterior sections of the medial and inferior temporal gyri have been associated with semantic control abilities across different sensory modalities [Hodges et al. 1992; Vandenberghe et al. 1996; Indefrey and Levelt 2004; Vigneau et al. 2006; Binder et al. 2009; Whitney et al. 2011; Ralph et al. 2017]. A meta‐analysis further strengthened this view and highlighted the involvement of several left‐lateralized fronto‐temporal brain regions in semantic‐control processes, but also of the right prefrontal regions, including the right IFG [Jackson 2021]. In agreement, the right IFG has been repeatedly associated with non‐verbal control tasks (e.g., Stop Signal, see Hannah et al. 2020; Jana et al. 2020; Schaum et al. 2021; Sundby, Jana, and Aron 2021; Swann et al. 2009; Wessel et al. 2013), highlighting the possible domain‐general contribution of this frontal brain region for control mechanisms.

Although the above‐mentioned fMRI studies have contributed to the characterization of the brain regions involved in language control mechanisms using classical whole‐brain or ROI‐based approaches, the indirect nature of the fMRI recordings and its poor temporal resolution (several seconds) have prevented a thorough investigation of language control brain dynamics. As magnetoencephalography (MEG) provides a direct measure of neural activity with millisecond (ms) time resolution and good spatial reconstruction, it allows enhanced investigation of language control–related brain processes. In particular, MEG provides a precise reconstruction of language‐related brain activity within specific frequency bands that have been identified as critical for language processes, such as the beta‐band frequency (12–30 Hz) [Findlay et al. 2012; Fisher et al. 2008; Kadis et al. 2008; Kadis et al. 2011; Pang et al. 2011; Ressel et al. 2008; Youssofzadeh, Williamson, and Kadis 2017]. For instance, previous studies on verb generation have shown significant event‐related beta‐band (12–30 Hz) desynchronization (ERD) or suppression occurring approximately 300–800 ms, upon confrontation with a visually presented image, in fronto‐temporal regions of the language dominant hemisphere in both healthy subjects [Findlay et al. 2012; Fisher et al. 2008; Kadis et al. 2008; Kadis et al. 2011; Pang et al. 2011; Ressel et al. 2008; Youssofzadeh, Williamson, and Kadis 2017] and neurosurgical candidates [Findlay et al. 2012; Fisher et al. 2008; Hinkley et al. 2020; Youssofzadeh and Babajani‐Feremi 2019]. While the observed beta‐band ERD processes are relevant to verb generation and language processing, their contribution to language control processes falls outside the scope of these studies. Instead, it has been suggested that beta‐band event‐related synchronization (ERS) or enhancement are particularly involved in control mechanisms. Accordingly, a recent MEG study showed that event‐related beta‐band (12–31 Hz) ERS, occurring between 100 and 350 ms after stimulus presentation, was associated with non‐verbal cognitive control mechanisms in the context of a Stop‐Signal task [Schaum et al. 2021]. At the cognitive level, the classical interpretation suggests that ERS serves as a possible mechanism that elicits inhibition and suppression of irrelevant information [Pfurtscheller 1992; Pfurtscheller, Stancák, and Neuper 1996; Pfurtscheller and Andrew 1999]. This is in line with the classical interpretation of ERS as reflecting, at the neuronal level, a decrease of cortical excitability or a cortical inhibition, and particularly in the beta and alpha bands [Pfurtscheller 2003] as this interpretation does not generalize to ERS phenomena in all frequency bands. Indeed, gamma and theta ERS have classically been linked to increased cortical excitability (for gamma‐band, see Crone et al. 1998; Miller et al. 2007; Pfurtscheller et al. 1997; for theta‐band see, Aftanas et al. 2001; Aftanas et al. 2002; Balconi and Lucchiari 2006; Knyazev, Slobodskoj‐Plusnin, and Bocharov 2009; Mu et al. 2008). In parallel, ERD processes were often observed in the context of more automatic or less controlled conditions, and suggested to reflect increased cortical excitability/activation and enhanced information processing [Pfurtscheller 1992; Pfurtscheller, Stancák, and Neuper 1996; Pfurtscheller and Andrew 1999; Schaum et al. 2021].

Hence, investigating the temporal and spectral properties of brain synchronization processes appears critical to better understand language control processes. To the best of our knowledge, only one electroencephalographic (EEG, 64 electrodes) study has investigated verbal cognitive control mechanisms and reported an association between early beta‐band ERS processes and verbal inhibition [Castiglione et al. 2019]. Yet, this study did not focus on language control processes *per se* (i.e., participants had to inhibit the retrieval of the second word of a newly learned pair of words) and source reconstruction analyses were not reported, preventing the characterization of the brain regions associated with these phenomena. The present study fills this gap by characterizing the spatio‐temporal dynamics of brain synchronization mechanisms associated with lexical–semantic control processes involved in language production, using both VGEN and PN tasks with two levels of lexical–semantic control (HC and LC conditions). Based on previous literature suggesting an association between early beta‐band ERS processes and verbal [Castiglione et al. 2019], as well as non‐verbal, cognitive control mechanisms [Hannah et al. 2020; Jana et al. 2020; Schaum et al. 2021; Sundby, Jana, and Aron 2021; Swann et al. 2009; Wessel et al. 2013], we hypothesized that HC task conditions requiring higher levels of lexical–semantic control processes (HC>LC) would be associated with more pronounced early beta‐band ERS in a set of fronto‐temporal brain areas previously reported for being involved in lexical and/or semantic control brain processes [Jackson 2021]. We additionally expected these effects to be more pronounced in the context of the VGEN task than in the PN task. Participants also underwent fMRI investigations using similar experimental paradigms (but different verbal material) to complement our MEG findings and previous fMRI results.

## 2 Materials and Methods

### 2.1 Participants

Thirty right‐handed, native French healthy adults (16 females, 14 males; median age: 30 years, 25th percentile: 25 years, 75th percentile: 36 years, age range: 22–55 years old) gave written informed consent to participate in this experiment approved by the Biomedical Ethics Committee of the CUB—Hôpital Erasme (EudraCT/CCB: B406201732244; Reference: P2017/272)—Université libre de Bruxelles (ULB). None of them had prior history of neurological or language disorders, nor any contra‐indication for MEG or MRI. According to the Edinburgh Handedness Inventory [Oldfield 1971], all of them were right‐handed (median laterality index = 100, 25th percentile = 86.50, 75th percentile = 100, range: 77.77–100).

### 2.2 Materials and Preliminary Behavioral Study

The experimental material used in this study was adapted from Thompson‐Schill, D'Esposito, Aguirre, and Farah [1997] into a French language version and using colored pictures of objects. 160 pictures of objects were taken from the Rossion and Pourtois [2004] database and assigned to VGEN task, and another set of 160 pictures of objects were taken from the MultiPic [Duñabeitia et al. 2018] for PN task.

A pilot behavioral study was conducted to validate the experimental material in a separate group of 68 participants (35 females, 33 males; median age: 23 years, 25th percentile: 21 years, 75th percentile: 26 years, age range: 19–43 years), who were asked to overtly generate the first single verb (VGEN task) or noun (PN task) that came to their mind upon confrontation with the pictures. A response strength ratio was then computed for each item (i.e., the relative frequency of the most common answer to the relative frequency of the second‐most common answer; according to Thompson‐Schill, D'Esposito, Aguirre, and Farah [1997]), and determined which items were assigned to the LC (high ratio, range 5.00–50.00, median 10.00) or HC (low ratio, range 1.00–3.00, median 1.58) condition. Half items were classified as HC and the other half as LC. A Mann–Whitney U test revealed a significant difference between response strength ratios in the LC and the HC conditions (VGEN: U = 6370, z = 10.82, *p* < 0.001; PN: U = 6399, z = 10.92, *p* < 0.001). No significant difference was found between the stimuli from both conditions (HC vs. LC) in terms of word frequency (VGEN: U = 3042, z = −0.001, *p* = 0.99; PN: U = 2796, z = −0.99, *p* = 0.31), visual complexity (VGEN: U = 2661, z = −1.83, *p* = 0.6; PN: U = 2882, z = −1.08, *p* = 0.27), and familiarity (VGEN: U = 3439, z = 0.81, *p* = 0.41; PN: U = 3372, z = 0.58, *p* = 0.55). To complement these findings, Bayesian rank‐based tests were conducted to compare the HC and LC conditions for word frequency, visual complexity, and familiarity in both the VGEN and PN tasks. The Bayes factors (BF₀₁) provided additional evidence supporting the absence of substantial differences between conditions for these variables. Specifically, for the VGEN task, word frequency (BF₀₁ = 5.776), visual complexity (BF₀₁ = 1.829), and familiarity (BF₀₁ = 7.172) showed moderate to strong evidence in favor of the null hypothesis, suggesting no substantial difference between conditions. Similarly, for the PN task, word frequency (BF₀₁ = 6.374), visual complexity (BF₀₁ = 4.084), and familiarity (BF₀₁ = 6.206) indicated that the stimuli were well‐matched across these dimensions. The word frequency, visual complexity, and familiarity values were obtained from the Alario and Ferrand [1999] database. The median and percentiles (raw values) for word frequency, visual complexity, and familiarity in both conditions for tasks are provided in **Supplementary Table** 1. Moreover, items were attributed to one of the following semantic categories: animals, games, nature, body parts, food/beverages, tools, garments, household.

Both the VGEN and the PN tasks included 160 pictures that were divided into two sets of 80 images randomly assigned to the MEG or fMRI paradigms. The stimuli did not differ in terms of presentation parameters and duration.

### 2.3 MEG/fMRI Tasks and Protocol

MEG recordings were performed before fMRI in all subjects to avoid any risk of participants' magnetization. Prior to entering the MEG, participants underwent a short practice session on a separate set of images to get familiar with the timing of the stimuli presentation and to ensure that they properly understood the VGEN and PN tasks while they were asked to overtly perform them for five trial items per task.

**FIGURE 1:**
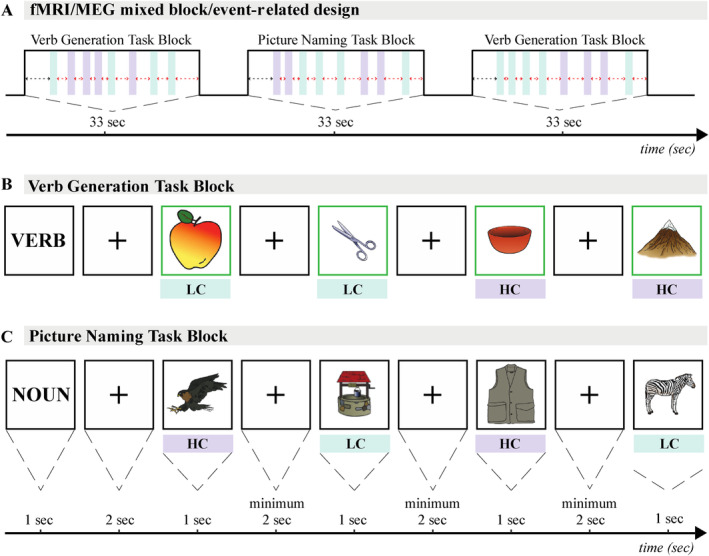
Schematic representation of the fast‐mixed block/event‐related MEG fMRI design. A. Mixed block/event‐related design. Each participant underwent alternating 33‐s blocks of covert picture naming (PN) or verb generation (VGEN) (order randomized per participant) for a total of 20 blocks. B. Example of VGEN protocol. C. Example of PN protocol. According to the task, participants were asked to covertly generate a single verb (VGEN) or noun (PN) that came to their mind upon confrontation with pictures requiring low (LC) or high (HC) lexical–semantic control.

Participants were then asked to silently perform the VGEN and the PN tasks (occurring in the same experimental conditions). During data acquisition, participants were presented with alternating 33‐s blocks of VGEN and PN tasks (Figure 1), for a total of 20 blocks (i.e., 10 VGEN and 10 PN blocks), starting with VGEN or PN depending on a per participant randomization. Each block started with a cue indicating the type of task to follow (VGEN: VERB, PN: NOUN, 1 s duration followed by a fixation cross of 2 s). Each block was separated by a period of rest of variable duration (from 2 to 7 s, mean = 2.75 s). Each block included 8 pictures of different objects randomly selected among the 80 items (40 LC, 40 HC) attributed to the MEG or fMRI protocol. Of note, two pictures of the same object category, e.g., animals, were never displayed consecutively to avoid semantic priming effects [Hänze and Hesse 1993]. Each picture was displayed for 1 s and followed by a fixation cross with a varying inter‐stimulus interval (ISI) to reduce anticipation of the next trial (Figure 1B,C). The ISI was determined using the Optseq2 toolbox (http://surfer.nmr.mgh.harvard.edu/optseq; RRID: RRID:SCR_014363) in a stochastic design framework with the constraints that (i) each block had to last 33 s, (ii) each image had to be presented during 1 s, and (iii) fixation crosses between images had to last at least for 2 s. On average, fixation crosses between images were presented during 2.71 ± 0.80 s (range: 2–7 s) and mean rest periods between blocks had a duration of 3.07 ± 1.13 s (range: 2–6.5 s). This resulted in a mixed block/event‐related design.

### 2.4 fMRI Data Acquisition, Preprocessing, and Analyses

**fMRI data acquisition**. MRI data were always acquired after MEG data on a hybrid 3 Tesla SIGNA PET‐MR scanner (GE Healthcare, Milwaukee, Wisconsin, USA) with a 24‐channel head and neck coil (Nova Medical 3 T 32 Channel Head Coil, Nova Medical, Wilmington, USA).

Participants were comfortably installed in a supine position, with ear protection (ear plugs and headphones) to reduce acoustic noise. The experiment was programed and conducted using Psychtoolbox (version 3.0.14) implemented in Matlab (Mathworks Inc., Sherbom, MA, version 9.5). Stimuli were delivered and displayed on the MRI‐compatible Inroom Viewing Device (4 K LCD screen, version 3.0.4; NordicNeuroLab; NNL, Bergen, Norway) made visible to the participants through a mirror attached to the head coil, so that their appearance matched the MEG recording conditions (visual angle < 7°, see below).

fMRI data were acquired using a single‐shot gradient‐echo echo‐planar‐imaging (GE/EPI) T2*‐weighted images with whole‐brain coverage (time of repetition (TR) = 3000 ms, time of echo (TE) = 35 ms, flip angle (FA) = 90°, field of view (FOV) = 28 cm, matrix size = 96 x 96, in‐plane resolution = 2.7 x 2.7 mm, slice thickness = 3 mm, ascending interleaved acquisition, 43 slices). Four dummy scans were performed prior to data collection to allow the signal to reach a steady state. A structural 3D T1‐weighted sequence of the head was also acquired for each participant after fMRI (TR = 8.2 ms, TE = 3.1 ms, FA = 12°, FOV = 24 cm, matrix size = 240 x 240, isotropic 1 mm^3^ voxels).

**Preprocessing**. fMRI data were preprocessed using SPM12 (Wellcome Department of Cognitive Neurology, Institute of Neurology, London, UK, RRID:SCR_007037). Slice‐timing correction was first applied to functional images that were then realigned, co‐registered to structural images, normalized to the Montreal Neurological Institute (MNI) space based on the structural segmentation, and finally smoothed with a Gaussian filter (full width at half maximum = 8 mm).

**Statistical analyses**. fMRI analyses aimed at replicating previous results, in particular, those using similar experiments on lexical–semantic control [Thompson‐Schill, D'Esposito, Aguirre, and Farah 1997; Thompson‐Schill et al. 1998; Thompson‐Schill, D'Esposito, and Kan 1999], and thus confirm our neuroimaging protocol for MEG investigations.

To do so, whole‐brain individual functional maps were calculated by convolving four separate regressors [i.e., VGEN(LC), VGEN(HC), PN(LC), PN(HC)] with the canonical hemodynamic response function and its temporal and spatial derivates in a first‐level general linear model [Friston et al. 1994], while regressing out the movement parameters estimated at the realignment step. Individual maps associated with the aforementioned regressors were obtained by performing mass‐univariate, two‐sided, one‐sample t‐tests searching for brain areas showing a significant increase or decrease in the blood level oxygen dependent (BOLD) signal during HC and LC conditions in the VGEN and PN tasks separately compared with rest. Likewise, the main effect of HC vs. LC conditions was examined in each task separately. The effect of tasks (VGEN vs. PN) was also examined (across experimental conditions).

Group whole‐brain BOLD signals in HC and LC conditions were then statistically compared in the VGEN and PN tasks separately using mass‐univariate, two‐sided, paired t‐tests. The resulting t maps were first statistically thresholded with familywise error (FWE) rate controlled using random field theory (*p* ^FWE corr.^ < 0.05) [Nichols 2012]. If no significant finding was observed at FWE, we also set the significance threshold at *p* ^uncorr.^ < 0.001 at the voxel level. The uncorrected threshold was considered in this study given the expected low statistical significance based on the pervious findings from Thompson‐Schill, D'Esposito, Aguirre, and Farah [1997, 1998] and Thompson‐Schill, D'Esposito, and Kan [1999], which showed that the FWE rate correction is too stringent for investigating the effect of high vs. low lexical–semantic control brain processes on BOLD signal within the VGEN task. For the sake of clarity, the main effect of each lexical–semantic control condition (i.e., HC vs. rest and LC vs. rest) was also examined in each task as well as the effect of tasks (VGEN vs. PN) across experimental conditions.

### 2.5 MEG Data Acquisition, Preprocessing, and Analysis

**MEG data acquisition**. As for fMRI, the MEG experiment was programed and launched using Psychtoolbox (version 3.0.14). Stimuli were projected onto a back‐projection screen placed vertically, approximately 120 cm away from the MEG helmet. Participants were installed in a supine position and could see the screen through a mirror placed above their head. The optical path from the screen to participants' eyes was approximately 140 cm (visual angle < 7°, object size = 17 cm).

MEG data were recorded using a simultaneous 306‐channel MEG (Triux, MEGIN, Finland) placed in a lightweight magnetically shielded room. This system includes 102 sensor triplets containing one magnetometer and two orthogonal planar gradiometers. Magnetometers measure the radial component of the magnetic field, while planar gradiometers measure its spatial derivative in tangential directions. The MEG signal was acquired with an analog band‐pass filter at 0.1–330 Hz and a 1 kHz sampling rate. Four head‐position indicator coils were used to continuously monitor participants' head position. The location of these coils and of at least 400 head‐surface points (on scalp, nose, and face) relative to anatomical fiducials were digitized with an electromagnetic tracker (Fastrak, Polhemus, Colchester, Vermont, USA).

**Preprocessing**. MEG data were first preprocessed off‐line using signal space separation (SSS) algorithm implemented in the MaxFilter software (version 2.2 with default parameters, MEGIN, Helsinki, Finland) to suppress external magnetic interferences and correct for head movements [Taulu, Simola, and Kajola 2004; Taulu and Simola 2006]. Then, an independent component analysis (FastICA algorithm with dimension reduction to 30 components; hyperbolic tangent nonlinearity function) was applied to the filtered MEG data (off‐line band‐pass filtered at 0.5–70 Hz; with a notch filter at 48–52 Hz) to identify physiological (i.e., heartbeats, eye‐blinks, and eye‐movements) and electromagnetic artifacts [Vigário et al. 2000]. Components corresponding to artifacts were visually identified and removed from the initial MEG data, i.e., prior to band‐filtering and dimension reduction, by spatial regression of their topographies. Across all subjects and all experimental conditions, the mean ± SD number of subtracted components from data was 2.17 ± 0.59 (range: 1–4). Cleaned MEG data were then epoched from −2 s to 3 s relative to the onset of the stimulus (picture) presentation. Epochs during which at least one magnetometer exceeded 3pT or at least one gradiometer exceeded 70pT/m were rejected [Bourguignon et al. 2011]. A two‐way repeated‐measure ANOVA performed on the number of artifact‐free epochs did not reveal any effect of task (VGEN vs. PN, *p* = 0.93), or lexical–semantic control condition (HC vs. LC, *p* = 0.74), nor any interaction (*p* = 0.44). In addition, a two‐way repeated measures Bayesian ANOVA examined the effects of task, lexical–semantic control condition, and their interaction. The Bayes factors (BF₀₁) for task (BF₀₁ = 3.507), condition (BF₀₁ = 3.782), and interaction (BF₀₁ = 24.782) indicated moderate to strong support for the null hypothesis, suggesting that neither task, condition, nor their interaction had a significant effect on the number of artifact‐free epochs. In each condition (HC or LC), there were a total of 40 trials before artifact rejection. After rejecting artifacts, Mann–Whitney U tests showed no significant difference in the number of trials between the HC and LC conditions in both tasks. In the VGEN task, the LC condition had a median of 38 (25th percentile = 37.25, 75th percentile = 38), and the HC condition had a median of 38 (25th percentile = 37, 75th percentile = 38) (U = 518, z = 1.14, *p* = 0.25). In the PN task, the LC condition had a median of 38 (25th percentile = 37, 75th percentile = 38), and the HC condition had a median of 38 (25th percentile = 38, 75th percentile = 38) (U = 396, z = −0.93, *p* = 0.35). Bayesian rank‐based tests further supported these findings. For the VGEN task, the Bayes factor (BF₀₁ = 3.406) provided moderate evidence in favor of the null hypothesis, while for the PN task, the Bayes factor (BF₀₁ = 4.735) provided strong evidence for the null hypothesis, indicating no substantial differences between HC and LC conditions.

**Time‐frequency decomposition**. Epoched MEG sensor signals were decomposed spectrally using Morlet wavelet analysis (from −500 ms to 1000 ms peristimulus, frequency range 12–30 Hz with a 1 Hz step, 7‐cycle Morlet wavelets) as implemented in Fieldtrip [Oostenveld et al. 2011]. This decomposition was restricted to the beta‐band (12–30 Hz) based on its demonstrated involvement in verbal [Castiglione et al. 2019] and non‐verbal [Hannah et al. 2020; Jana et al. 2020; Schaum et al. 2021; Sundby, Jana, and Aron 2021; Swann et al. 2009; Wessel et al. 2013] cognitive control tasks.

**Source reconstruction**. Head modeling was computed using a one‐layer Boundary Element Method (MNE‐C software suite, Gramfort et al. 2014; Martinos Center for Biomedical Imaging, MA, USA; RRID:SCR_005972) based on the inner skull surface derived from each adult's MRI tissue segmentation (FreeSurfer software, Fischl 2012; Martinos Center for Biomedical Imaging, MA, USA; RRID:SCR_001847). MEG and MRI coordinate systems were co‐registered using three anatomical fiducial points for initial estimation and the head‐surface points to manually refine the surface co‐registration. A 5‐mm cubic grid of dipole locations was built inside the inner skull volume of the Montreal Neurological Institute (MNI) space template and non‐linearly deformed onto each participant's MRI with Statistical Parametric Mapping (SPM12, Wellcome Department of Cognitive Neurology, London, UK).

The spectrally‐decomposed MEG activity was then source projected using Minimum Norm Estimation (MNE; Dale and Sereno 1993), which addresses the ill‐posed nature of MEG source estimation through regularization. The noise covariance matrix was estimated based on 5 minutes of empty‐room data preprocessed with SSS, filtered between 0.1–70 Hz including a 50 Hz notch filter to account for line noise. Wideband noise data were used to ensure the inverse model appropriately covers the frequency range analyzed in the Morlet wavelet decomposition. The MNE regularization parameter was based on the consistency condition [Wens et al. 2015] and the MNE depth bias was corrected through a noise standardization scheme, i.e., sLORETA [Pascual‐Marqui 2002].

**Spectral power analyses**. Spectral power was estimated as the magnitude squared of the source‐projected Morlet wavelet coefficients. Source ERD/ERS values were then computed as the relative difference between post‐stimulus power and pre‐stimulus power averaged in the baseline ‐400 ms to ‐100 ms. Post‐stimulus power decreases (compared to a pre‐stimulus baseline) of source oscillatory power in specific frequency bands were identified as ERD, and increases as ERS [Pfurtscheller 2001]. In order to take inter‐subject variability into account [Holmes and Friston 1998], ERD/ERS time‐frequency data were then computed for each subject, and each lexical–semantic control condition (HC; LC).

**Statistical analyses**. Resulting source ERD/ERS associated with HC and LC trials were statistically compared (i) separately to the baseline period (i.e., HC or LC vs. baseline) using mass‐univariate, two‐sided, one‐sample t‐tests, and (ii) to each other (HC vs. LC) using mass‐univariate, two‐sided, paired t‐tests. Given our hypothesis that MEG would enhance our ability to differentiate the functional brain processes underlying high and low lexical–semantic control demands, we applied a threshold of *p* ^FWE corr.^ < 0.05 to the HC vs. LC contrasts. Each comparison led to statistical time‐frequency brain maps. Statistical significance was set to *p* ^FWE corr.^ < 0.05 with the FWE rate in the whole‐brain source space controlled by the Bonferroni correction of the number of spatial degrees of freedom available in MNE reconstructions [Wens et al. 2015], quite analogously to fMRI random field theory [Worsley et al. 1996]. Of note, applying a spatial (not temporal, see the discussion section for associated limitations) partial multiple comparison correction to the MEG data allowed having comparable statistical sensitivities between the MEG/fMRI data analyses avoiding a bias in favor of fMRI, which did not suffer any temporal comparisons.

To better characterize the spatial signature of ERD/ERS processes associated with lexical–semantic control mechanisms, brain maps of significant ERD/ERS were constructed in two beta‐bands, considering the distinctive roles of low beta (12–20 Hz) and high beta (21–30 Hz) frequency bands in verbal and non‐verbal control processes (e.g., Castiglione et al. 2019; Schaum et al. 2021). This was done by averaging, for each source separately, all supra‐threshold t‐values over the corresponding band and over successive 0.1‐s long‐time windows. To characterize associated dynamics, the time courses were extracted at the local maxima of significant ERD/ERS brain maps.

## 3 Results

### 3.1 fMRI Results

**FIGURE 2:**
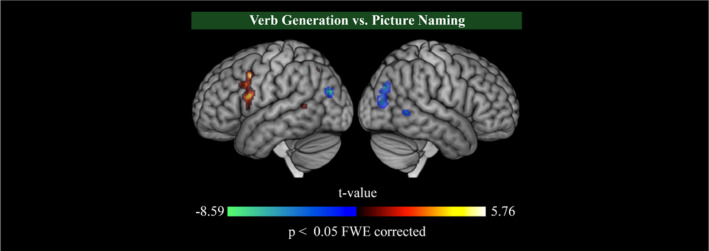
Whole‐brain group‐level statistical parametric maps for the effect of tasks. Mean fMRI activation map showing significantly higher increase (positive response; red) or decrease (negative response; blue) of the blood level oxygen dependent (BOLD) signal in the VGEN compared to PN task (*p* ^FWE corr.^ < 0.05).

fMRI analyses investigating the effect of tasks (i.e., VGEN vs. PN) revealed a significantly higher increase (VGEN>PN, *p* ^FWE corr.^ < 0.05) in BOLD signal in the left inferior frontal gyrus ([−50, 10, 18] mm; t‐value = 5.76), the left precentral gyrus ([−42, 8, 42] mm; t‐value = 5.55), the left middle temporal gyrus ([−56, −52, 8] mm; t‐value = 4.99), and the left angular gyrus ([−56, −52, 8] mm; t‐value = 4.99) during VGEN compared with PN. The BOLD signal was significantly higher during PN compared with VGEN (PN>VGEN, *p* ^FWE corr.^ < 0.05) in more posterior brain areas, and particularly in bilateral occipital areas (left [−34, −82, 24] mm; t‐value = −8.59; right [34, 76, 30] mm; t‐value = −8.05) (Figure 2).

Examining the main effect of each lexical–semantic control condition (i.e., HC vs. rest and LC vs. rest), fMRI analyses showed that HC were characterized by significant increases in BOLD signal (*p* ^FWE corr.^ < 0.05) in a set of fronto‐temporal brain areas, and only in the VGEN task (see **Supplementary Figures** S1).

**FIGURE 3:**
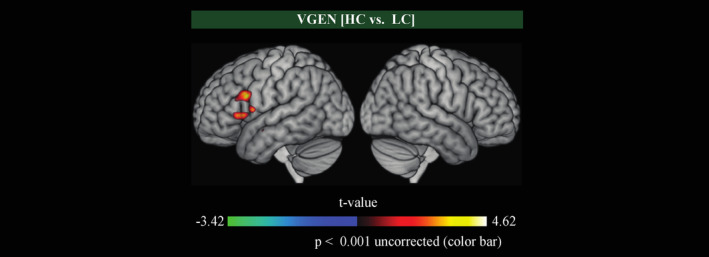
Whole‐brain group‐level statistical parametric maps for the VGEN task. Mean fMRI activation map showing increase (positive response; red) or decrease (negative response; blue) of blood level oxygen dependent (BOLD) signal in the HC compared with the LC condition. The color scale corresponds to voxel t‐values and the map is masked statistically at *p* ^uncorr.^ < 0.001. No voxel survived correction for the familywise error (FWE) rate using random field theory (*p* ^FWE corr.^ < 0.05).

fMRI analyses investigating the effect of high vs. low lexical–semantic control brain processes on BOLD signal within the VGEN task revealed significant differences of brain activity after running a comparison at a lower threshold (*p* ^uncorr.^ < 0.001), which replicates previous results from Thompson‐Schill, D'Esposito, Aguirre, and Farah [1997, 1998] and Thompson‐Schill, D'Esposito, and Kan [1999]. Our results showed HC>LC‐dependent modulations of BOLD signal in the left inferior frontal gyrus ([−52, 14, 24] mm; t‐value = 4.35) in the VGEN task (Figure 3). Finally, no significant differences were observed in the context of the PN task (all *p* ^uncorr.^ > 0.001).

### 3.2 MEG Results

#### 3.2.1 Verb Generation Task: High vs. Low Lexical–Semantic Control Brain Processes

Based on the fMRI results, subsequent MEG analyses aiming at investigating the spatio‐temporal dynamics of brain synchronization processes involved in lexical–semantic control were only conducted on VGEN task–related data.

**FIGURE 4:**
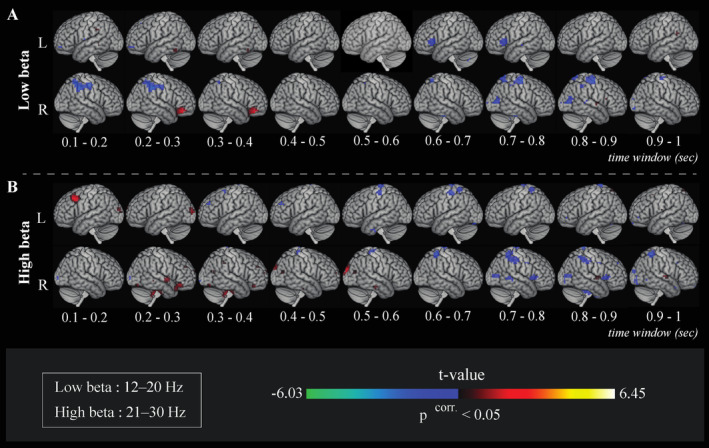
Brain maps displaying, for the verb generation (VGEN) trials, source locations showing significantly more pronounced power modulation of brain processes in the high compared with the low lexical–semantic control condition (HC vs. LC, *p* ^FWE corr.^ < 0.05) in the beta‐band (A. low beta; B. high beta) from 0.1 s. to 1 s post‐stimulus presentation. For HC compared with the LC condition, relative more pronounced desynchronizations (ERD) in low or high beta are coded in a blue to green scale, while relative more pronounced synchronizations (ERS and/or less pronounced ERD) are coded in a dark red to yellow scale.

MEG analyses performed on VGEN trials revealed significant differences of source beta‐band ERD/ERS between the high vs. low lexical–semantic control (HC vs. LC) conditions occurring from 0.1 s up to 1 s post‐stimulus onset in a distributed set of cerebral regions (*p* ^FWE corr.^ < 0.05; Figure 4 and **Supplementary Table** 2 for details on local maxima across time and space).

**FIGURE 5:**
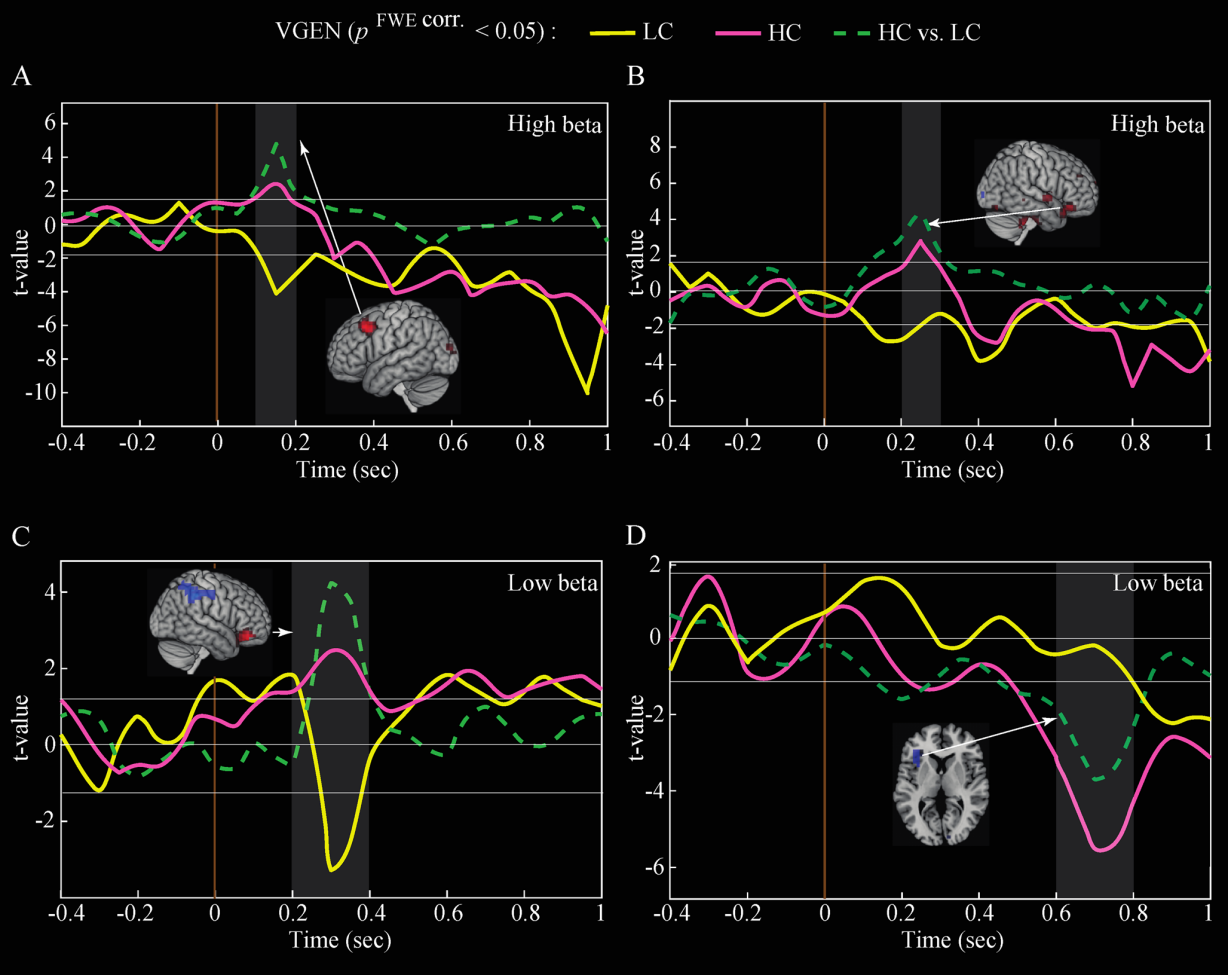
Time courses of statistical t‐values of ERS/ERD in the high beta (higher panel) and low beta (lower panel) frequency bands, in brain regions showing a more pronounced modulation in the HC compared with the LC condition (*p* ^FWE corr.^ < 0.05) during the VGEN task. HC>LC‐related brain processes associated with less pronounced ERD (compared with the LC condition) in: (A) the left dorsolateral prefrontal cortex (0.1–0.2 s), (B) the right inferior frontal gyrus (0.2–0.3 s) in the high beta‐band (high panel), and (C) the right inferior frontal gyrus (0.2–0.4 s) in the low beta‐band (low panel). (D) HC>LC‐related brain processes associated with more pronounced ERD (compared with the LC condition) in the left insula between 0.6–0.8 s (low panel).

In the early time windows (0.1–0.2 s), more pronounced ERD was found in the HC condition compared with the LC condition in the right supramarginal gyrus ([42, −40, 43] mm; t‐value = −4.14) and the left inferior frontal gyrus ([−35, 56, −11] mm; t‐value = −3.69) in the low beta‐band. From 0.2–0.4 s, compared with the LC condition, the HC condition was associated with more pronounced ERD in the right supramarginal gyrus ([42, −40, 43] mm; t‐value = −4.24) and the left inferior frontal gyrus ([−34, 58, −11] mm; t‐value = −3.69) in the low beta‐band, as well as with more pronounced ERD in the left dorsolateral prefrontal cortex ([−40, 45, 31] mm; t‐value = −3.78) in the high beta‐band. Worth noticing, the analysis of the temporal courses of power amplitudes associated with the high and low lexical–semantic control conditions, revealed that all positive contrasts (i.e., HC>LC) corresponded to a less pronounced ERD in the HC as compared with the LC condition, rather than an actual increase of ERS amplitude in beta‐band power during the HC condition. More precisely, less pronounced ERD were observed between 0.1–0.2 s in the left dorsolateral prefrontal cortex ([−37, 17, 44] mm; t‐value = 4.63) in the high beta‐band (Figure 5A, high panel), as well as from 0.2–0.4 s in the right inferior frontal gyrus (high beta [45, 28, −17] mm; t‐value = 4.05; low beta [34, 34, −15] mm; t‐value = 4.39) (Figure 5B,C, high and low panel).

In the later time‐windows (0.6–0.8 s), a more pronounced HC>LC ERD (*p* ^FWE corr.^ < 0.05) was observed in the low and high beta‐bands within a set of mainly right‐lateralized fronto‐temporo‐parietal regions, including the right inferior frontal gyrus ([42, 18, 4] mm; t‐value = −3.83), the right middle temporal gyrus ([67, −37, −4] mm; t‐value = −3.67), and the right superior parietal gyrus ([39, −47, 63] mm; t‐value = −3.98). More pronounced low beta power ERD were also found in the HC (as compared with LC) condition in the left insula ([−36, 24, 4] mm; t‐value = −3.95) (**Supplementary Table** 2 for details on local maxima across time and space).

In addition, the analysis of power amplitude–related temporal courses associated with the HC and LC conditions revealed distinct temporal patterns in the high and low beta synchronization processes. Specifically, low beta exhibited an early (0.2–0.4 s) less pronounced ERD in the right inferior frontal gyrus (Figure 5C), followed by a later (0.6–0.8 s), more pronounced ERD in the left insula for the HC>LC‐related brain processes (Figure 5D). In contrast, high beta displayed a less pronounced ERD early on in both the left dorsolateral prefrontal cortex (0.1–0.2 s; Figure 5A) and the right inferior frontal gyrus (0.2–0.3 s; Figure 5B) in the HC condition as compared to LC condition.

For the sake of completeness, MEG results comparing HC and LC trials separately to baseline (i.e., HC or LC vs. baseline) in the beta frequency bands are reported **in Supplementary Figures** S2 and S3.

## 4 Discussion

The current study aimed at investigating the oscillatory brain dynamics underlying language control processes, a mechanism that critically allows the flexible access to context‐relevant representations during language production tasks. To do so, we recorded functional brain activity related to high (HC) and low (LC) lexical–semantic control conditions using fMRI and MEG in two language production tasks, namely a VGEN and a PN task.

fMRI analyses confirmed that the VGEN task, as compared to the PN task, elicited a stronger increase in BOLD signal in key left‐hemisphere brain regions previously associated with lexical or semantic control mechanisms, such as the left inferior frontal gyrus and the posterior middle temporal gyrus (see Bourguignon et al. 2018, and Jackson 2021 for a meta‐analysis). Furthermore, when investigating the effect of high vs. low lexical–semantic control brain processes on BOLD signal within the VGEN task, we replicated prior fMRI results obtained at a similar statistical threshold with similar lexical–semantic control tasks [Thompson‐Schill, D'Esposito, Aguirre, and Farah 1997; Thompson‐Schill et al. 1998; Thompson‐Schill, D'Esposito, and Kan 1999], therefore strengthening the relevance of our neuroimaging protocol for MEG investigations.

While our fMRI results revealed significant involvement of posterior temporal areas, particularly when contrasting VGEN to PN task, we did not observe significant posterior temporal neural modulation in our MEG analysis. Each modality has its own limitations and potential disadvantages. fMRI and MEG are sensitive to different types of neuronal activity. Specifically, non‐synchronous neuronal activity might lead to significant BOLD signal modulations in fMRI without detectable signals in MEG recordings [Kujala et al. 2014]. Additionally, transient neural patterns picked up by MEG may not be reflected in the hemodynamic signal captured by fMRI [Kujala et al. 2014].

MEG results focusing on the VGEN task revealed that, compared to the LC condition, the HC condition was associated with a sequence of beta‐band brain (de)synchronization processes (ERD/ERS) within a set of fronto‐temporo‐parieto‐occipital brain regions. Among these results, we observed a modulation of beta‐band (de)synchronization processes from 100 to 400 ms and from 600 to 900 ms post‐stimulus onset within frontal brain regions. In particular, while the HC (as compared with LC) condition was associated with less pronounced early beta‐band ERD in the left dorsolateral prefrontal cortex (100 ms) and right inferior frontal (200–400 ms) regions, beta‐band ERD mechanisms were bilaterally identified in the IFG at later stages (600–900 ms).

It is important to note that the observed early less pronounced ERD in the beta‐band contrasts with previous studies indicating an early involvement (< 0.4 s) of beta‐band related ERS mechanisms within right frontal regions in the context of verbal [Castiglione et al. 2019] or non‐verbal [Hannah et al. 2020; Jana et al. 2020; Schaum et al. 2021; Sundby, Jana, and Aron 2021; Swann et al. 2009; Wessel et al. 2013] cognitive control tasks (see also Noonan et al. 2013; Jackson 2021 for a discussion regarding the possible involvement of the right IFG in semantic control processes). Yet, as the previous studies did not analyze the temporal courses of power amplitude associated with their results, it remains possible that the previously reported early beta‐band ERS could, in fact, reflect, as in our data, a less pronounced ERD.

In our MEG study, we showed that an association between early less pronounced beta‐band ERD in *the right* IFG underlined language control processes, while previous fMRI studies reported a specific role of *the left* IFG toward lexical–semantic control mechanisms (as replicated in our fMRI results) [Thompson‐Schill, D'Esposito, Aguirre, and Farah 1997; Thompson‐Schill et al. 1998; Thompson‐Schill, D'Esposito, and Kan 1999]. In addition, results showed that all positive contrasts (i.e., HC>LC) corresponded to a less pronounced ERD in the HC as compared with the LC condition rather than an actual increase of ERS amplitude in beta‐band power. Altogether, these results highlighted the importance of analyzing the temporal courses of power amplitude to characterize language‐control processes as it is critical to interpret properly synchronization processes as well as it may reveal complementary results.

Our MEG results also help identifying significant early (< 0.3 s) HC>LC differences, and in particular, more pronounced early beta‐band ERD modulations in the right supramarginal gyrus, a region known to be involved in attentional processes [Ciaramelli, Grady, and Moscovitch 2008; Seghier 2013]. This result might thus reflect a boost of attentional resources associated with increased control demands to access and select context‐appropriate verbal representations [Krieger‐Redwood et al. 2015].

At later time courses (600–900 ms), MEG results additionally revealed a more pronounced modulation of right‐lateralized brain (de)synchronization processes within fronto‐temporo‐parietal regions (including the left IFG), in the high compared to the low lexical–semantic control condition. Articulation processes have been described to occur after 600 ms (see Indefrey and Levelt 2004, and Indefrey 2011 for meta‐analysis of language production) within these brain regions. However, VGEN‐related articulation processes should occur similarly in the low and high lexical–semantic control conditions, precluding an association between these results and articulation processes. However, beta‐related ERD occurring at these later time scales (from 600 to 900 ms) within the fronto‐temporo‐parietal brain regions may actually relate to control processes. Accordingly, the right fronto‐temporo‐parietal network and the left inferior frontal gyrus have been involved in verbal response monitoring and search for error [Ganushchak and Schiller 2008]. In that line, Levelt's perceptual loop theory [Levelt 1983; Levelt 1989] defines verbal monitoring processes as mechanisms controlling for the relevance of the response, the inspection of the speech plan, and the detection of errors before and after the response is articulated (covertly or overtly) [Levelt 1983; Levelt 1989]. Hence, later beta‐band ERD processes observed within the fronto‐temporo‐parietal brain regions in the HC condition could reflect a stronger recruitment of the controlled processes that are needed when multiple verbal lexical–semantic representations stored in long‐term memory enter into competition for selection (e.g., *play*, *kick*, *throw*, *dribble*, *hit*, *pass* for *ball*). In that line, several studies have reported late beta‐band (i.e.,12–30 Hz) ERD over the fronto‐temporo‐parietal brain regions in the context of semantic language paradigms comparing incongruent relative to congruent stimuli [Bastiaansen, Magyari, and Hagoort 2010; Luo et al. 2010; Wang et al. 2012]. Recent studies have suggested that the beta‐band can be subdivided into low beta (12–20 Hz) and high beta (21–30 Hz) ranges, with potentially distinct functional roles [Kopell, Whittington, and Kramer 2011; Roopun et al. 2008; Spitzer and Haegens 2017]. Low beta‐band ERD, in particular, has been linked to working memory processes, especially in tasks that require the retention and manipulation of verbal representations [Spitzer and Haegens 2017]. Thus, the later low beta‐band ERD observed in our study (**Supplementary Figure** S4) may not only reflect controlled verbal monitoring but also the involvement of working memory in maintaining and selecting competing lexical–semantic representations. These brain processes may thus help the controlled monitoring of internal representations during speech planning by verifying the potential mismatch between intended and actual verbal production. A tentative hypothesis could be that the early less pronounced ERD (i.e., indirectly observed as ERS in our analyses) within the right IFG from 200 to 400 ms post‐stimulus onset would allow the left homologous region to implement lexical–semantic control underlined by more pronounced ERD in the HC compared with the LC condition within the left IFG at later time scales (from 600 to 900 ms, post‐stimulus onset). This interpretation is highly compatible with the inter‐hemispheric balance theory originally proposed by Kinsbourne [1977], surmising that efficient cognitive processes would rely on the specialization of one (dominant) hemisphere in parallel with the inhibition of the non‐dominant hemisphere. Altogether, this may suggest that lexical–semantic control processes rely on a proper balance between the brain hemispheres, which is more important than the individual involvement of each hemisphere [Ríos‐López et al. 2020].

This study comprises two main limitations. At first, and as in several studies [Fisher et al. 2008; Kadis et al. 2008; Singh et al. 2002], we used a silent version of the VGEN task to avoid any contamination by muscle/movement artifacts associated with speech production in our data. Such approach did not allow us to characterize the behavioral responses of our participants. Also, it may be hypothesized that opting for a covert verbal production has decreased the signal‐to‐noise ratio in our fMRI and MEG measurements. Still, this hypothesis remains debatable since previous studies have reported highly consistent results using covert and overt versions of the VGEN task [Croft et al. 2013; Partovi et al. 2012; Vannest et al. 2010]. And finally, our study applied a partial multiple comparison correction to the MEG data, where the spatial comparison factor was corrected but not the temporal factor. This may have led to false‐positive associated with temporal comparison in our MEG analyses. Still, this approach was preferred as it enabled us to have comparable statistical sensitivities between our MEG/fMRI analyses, thus avoiding a possible bias in favor of fMRI (in which no temporal comparisons needed correction).

## 5 Conclusion

Altogether, this study strengthened the relevance of characterizing the spatio‐temporal dynamics of brain (de)synchronization associated with lexical–semantic control processes involved in language production. It showed that language control processes critically rely on a complex sequence of early and late brain synchronization (ERS/ERD) processes encompassing fronto‐temporo‐parietal brain regions, with the prefrontal areas being particularly involved at early stages (< 500 ms). These findings open new avenues of research regarding the specific role of associated prefrontal oscillatory mechanisms to the efficient selection of lexical–semantic context‐appropriate language representations in the human brain.

## Author Contributions

**Alexandru Mihai Dumitrescu:** conceptualization, formal analysis, investigation, writing – original draft, and visualization. **Tim Coolen:** conceptualization, software, formal analysis, investigation, writing – review and editing, and visualization. **Vincent Wens:** methodology, software, formal analysis, resources, and writing – review and editing. **Antonin Rovai:** resources, software. **Nicola Trotta:** resources, software. **Serge Goldman:** writing – review and editing, supervision, project administration, and funding acquisition. **Xavier De Tiège:** conceptualization, writing – review and editing, project administration, and funding acquisition. **Charline Urbain:** conceptualization, writing – review and editing, and supervision.

## Conflicts of Interest

The authors declare no conflicts of interest.

## Supporting information


**Data S1** Supporting Information

## Data Availability

The data that support the findings of this study are available from the corresponding author upon reasonable request.
