# Supplementary material for: Investigating the Spatio‐Temporal Signatures of Language Control–Related Brain Synchronization Processes
Source: Hum Brain Mapp. 2025 Jan 21;46(2):e70109. doi: 10.1002/hbm.70109 (PMC11747998; doi:10.1002/hbm.70109)
Supplement: Supplementary file 1 — Data S1 Supporting Information [file HBM-46-e70109-s001.docx]

Supplementary Material

**Investigating the spatio-temporal signatures of language control-related brain synchronization processes**

Alexandru Mihai DUMITRESCU^1*^, Tim COOLEN^1,2*^, Vincent WENS^1,3^, Antonin ROVAI^1,3^, Nicola TROTTA^1,3,4^, Serge GOLDMAN^1,3^, Xavier De TIÈGE^1,3^,

and Charline URBAIN^1,5^

*^1^* *Université libre de Bruxelles (ULB), UNI – ULB Neuroscience Institute, Laboratoire de Neuroanatomie et Neuroimagerie translationnelles (LN^2^T), Route de Lennik 808 1070 Bruxelles, Belgium*

*^2^ Université libre de Bruxelles (ULB), Hôpital Universitaire de Bruxelles (H.U.B.), CUB Hôpital Erasme, Department of Radiology, Route de Lennik 808 1070 Bruxelles, Belgium*

*^3^ Université libre de Bruxelles (ULB), Hôpital Universitaire de Bruxelles (H.U.B.), CUB Hôpital Erasme, Service of Translational Neuroimaging, Route de Lennik 808 1070 Bruxelles, Belgium*

*^4^ Université libre de Bruxelles (ULB), Hôpital Universitaire de Bruxelles (H.U.B.), CUB Hôpital Erasme, Department of Nuclear Medicine, Route de Lennik 808 1070 Bruxelles, Belgium*

*^5^ Université libre de Bruxelles (ULB), UNI – ULB Neuroscience Institute, Neuropsychology and Functional Neuroimaging Research Unit (UR2NF), Center for Research in Cognition and Neurosciences (CRCN), Avenue F.D, Roosevelt 50 1050 Bruxelles, Belgium*

** contributed equally to this study*

Corresponding author: Alexandru Mihai DUMITRESCU *-* Université libre de Bruxelles (ULB), UNI – ULB Neuroscience Institute, Laboratoire de Neuroanatomie et Neuroimagerie translationnelles (LN^2^T), Route de Lennik 808 1070 Bruxelles, Belgium. E-mail: [Alexandru.Mihai.Dumitrescu@ulb.be](mailto:Alexandru.Mihai.Dumitrescu@ulb.be)

| **Task** | **Linguistic measure** | **Condition** | **Median** | **P25** | **P75** |
| --- | --- | --- | --- | --- | --- |
| **VGEN** | *Word Frequency* | LC | 21.61 | 6.99 | 36.04 |
|  |  | HC | 30.02 | 10.93 | 64.8 |
|  | *Visual Complexity* | LC | 2.29 | 1.76 | 3.24 |
|  |  | HC | 2.55 | 2.13 | 3.56 |
|  | *Familiarity* | LC | 3.68 | 2.82 | 4.47 |
|  |  | HC | 3.76 | 2.58 | 4.37 |
| **PN** | *Word Frequency* | LC | 22.61 | 7.99 | 37.04 |
|  |  | HC | 29.32 | 10.01 | 63.87 |
|  | *Visual Complexity* | LC | 2.14 | 1.59 | 2.54 |
|  |  | HC | 2.54 | 1.72 | 2.93 |
|  | *Familiarity* | LC | 3.88 | 3.46 | 4.51 |
|  |  | HC | 4.01 | 3.38 | 4.34 |

**Supplementary Table 1.** Median as well as the 25th and 75th percentiles for word frequency, visual complexity, and familiarity in HC and LC for VGEN and PN task.

|  | **Low beta** | | | | | **High beta** | | | | | |
| --- | --- | --- | --- | --- | --- | --- | --- | --- | --- | --- | --- |
| **Time (sec)** | **MNI**  **[x y z]** | | | **Location** | **t-value** | **MNI**  **[x y z]** | | | **Location** | **t-value** |  |
| 0.1 - 0.2 | 64 | -40 | 35 | SMG | 3.77 | -37 | 17 | 44 | DLPFC | 4.63 |  |
|  | -6 | -89 | -10 | CAL | 3.70 | -26 | -95 | 16 | Omid | 3.88 |  |
|  | 42 | -40 | 43 | SMG(R) | -4.14 | 31 | -97 | 2 | Omid(R) | -3.64 |  |
|  | 42 | -18 | 47 | PrC(R) | -4.00 |  |  |  |  |  |  |
|  | -35 | 56 | -11 | LIFG | -3.69 |  |  |  |  |  |  |
| 0.2 - 0.3 | 34 | 34 | -15 | rIFG | 4.39 | 45 | 28 | -17 | rIFG | 4.05 |  |
|  | -41 | -54 | -17 | FUS | 3.95 | 37 | -34 | -39 | CBL(R) | 4.02 |  |
|  | -9 | -91 | -10 | CAL | 3.65 | -23 | -96 | 12 | Omid | 4.02 |  |
|  | 42 | -40 | 43 | SMG(R) | -4.24 | 64 | -3 | -3 | STG(R) | 3.94 |  |
|  | -34 | 58 | -11 | LIFG | -3.69 | 1 | 69 | 20 | SFGmid | 3.80 |  |
|  |  |  |  |  |  | 43 | 45 | 30 | DLPFC(R) | 3.56 |  |
|  |  |  |  |  |  | 31 | -97 | 2 | Omid(R) | -3.64 |  |
| 0.3 - 0.4 | 34 | 34 | -15 | rIFG | 4.19 | 37 | -33 | -38 | CBL(R) | 4.17 |  |
|  | -42 | -57 | -19 | FUS | 3.81 | 39 | 37 | -19 | rIFG | 3.93 |  |
|  | -45 | -75 | 40 | AG | 3.61 | 1 | 69 | 20 | SFGmid | 3.80 |  |
|  | 38 | -52 | 57 | SPG(R) | -3.70 | 55 | -67 | 18 | MTG(R) | 3.63 |  |
|  | -10 | 34 | 43 | SFGmid | -3.64 | 55 | -41 | 41 | SMG(R) | 3.61 |  |
|  |  |  |  |  |  | 43 | 45 | 30 | DLPFC(R) | 3.56 |  |
|  |  |  |  |  |  | -40 | 45 | 31 | DLPFC | -3.78 |  |
|  |  |  |  |  |  | 14 | -102 | -6 | CAL(R) | -3.77 |  |
| 0.4 - 0.5 | 26 | -92 | 15 | Osup(R) | 3.57 | 10 | -91 | 28 | Cun(R) | 4.05 |  |
|  | -31 | -62 | 67 | SPG | 3.56 | 34 | -74 | 18 | Omid(R) | 3.59 |  |
|  |  |  |  |  |  | -40 | 45 | 31 | MFG | -3.78 |  |
|  |  |  |  |  |  | 37 | -35 | 70 | PostC(R) | -3.57 |  |
| 0.5 - 0.6 | -18 | -79 | 2 | LG | 3.72 | 11 | -93 | 24 | Cun(R) | 4.55 |  |
|  | 32 | -47 | 53 | IPL(R) | -3.64 | 66 | -22 | -22 | ITG(R) | 3.89 |  |
|  |  |  |  |  |  | 34 | -74 | 18 | Omid(R) | 3.59 |  |
|  |  |  |  |  |  | -17 | -26 | 77 | PCL | -3.92 |  |
|  |  |  |  |  |  | -38 | -7 | -31 | ITG | -3.62 |  |
| 0.6 - 0.7 | -36 | 24 | 4 | INS | -3.95 | 16 | -92 | 25 | Osup(R) | 3.60 |  |
|  | 18 | -58 | 56 | SPG(R) | -3.71 | 39 | -47 | 63 | SPG(R) | -3.98 |  |
|  | -28 | -70 | -38 | CBL2 | -3.89 | -26 | -85 | -20 | CBL1 | -3.93 |  |
|  | -11 | -14 | -1 | TH | -3.69 | -30 | -23 | 48 | PrC | -3.84 |  |
|  |  |  |  |  |  | -2 | -28 | 78 | PCL | -3.77 |  |
|  |  |  |  |  |  | -34 | -47 | 67 | SPG | -3.72 |  |
|  |  |  |  |  |  | -18 | -68 | -6 | LG | -3.60 |  |
| 0.7 - 0.8 | -36 | 24 | 4 | INS | -3.93 | 17 | -74 | 17 | CAL(R) | -4.29 |  |
|  | 16 | -62 | 71 | SPG(R) | -3.88 | 40 | -46 | 57 | SPG(R) | -3.95 |  |
|  | 36 | -17 | 62 | PrC(R) | -3.84 | 39 | -30 | 39 | PostC(R) | -3.94 |  |
|  | 48 | -76 | 10 | MTG(R) | -3.81 | 42 | 18 | 4 | rIFG | -3.83 |  |
|  | -11 | -14 | -1 | TH | -3.69 | -2 | -28 | 78 | PCL | -3.77 |  |
|  | 11 | -97 | 5 | CAL(R) | -3.60 | 38 | -44 | 53 | IPL(R) | -3.77 |  |
|  |  |  |  |  |  | -34 | -47 | 67 | SPG | -3.72 |  |
|  |  |  |  |  |  | 67 | -37 | -4 | MTG(R) | -3.67 |  |
|  |  |  |  |  |  | 42 | -84 | 7 | Omid(R) | -3.66 |  |
|  |  |  |  |  |  | 52 | -74 | 8 | MTG(R) | -3.64 |  |
|  |  |  |  |  |  | -18 | -68 | -6 | LG | -3.60 |  |
| 0.8 - 0.9 | 64 | 19 | 15 | rIFG | 3.82 | 60 | -4 | 3 | STG(R) | 3.80 |  |
|  | 60 | -5 | 4 | STG(R) | 3.68 | -18 | -68 | 67 | SPG | 3.59 |  |
|  | -30 | 19 | -16 | INS | 3.53 | 16 | -38 | -15 | CBL(R) | -4.17 |  |
|  | 12 | -51 | 41 | PreCun(R) | -4.05 | 40 | -30 | 41 | PostC(R) | -4.11 |  |
|  | 37 | -18 | 62 | PrC(R) | -3.94 | -3 | -39 | 78 | PCL | -4.07 |  |
|  | 16 | -62 | 72 | SPG(R) | -3.90 | 42 | 18 | 4 | rIFG | -3.83 |  |
|  | 49 | -75 | 12 | MTG(R) | -3.86 | -45 | 49 | -6 | MFG | -3.72 |  |
|  | 13 | 12 | -96 | CAL® | -3.63 | 42 | -84 | 7 | Omid(R) | -3.66 |  |
|  | 45 | -44 | 48 | IPL(R) | -3.56 | 52 | -74 | 8 | MTG(R) | -3.64 |  |
| 0.9 - 1 | -60 | -53 | 26 | SMG | 3.85 | 62 | -12 | 4 | STG(R) | 3.90 |  |
|  | 19 | -89 | -8 | LG(R) | -3.82 | -18 | -68 | 67 | SPG | 3.59 |  |
|  | 38 | -23 | 68 | PrC(R) | -3.69 | -65 | -12 | 15 | PostC | 3.58 |  |
|  | 31 | -97 | 15 | Omid(R) | -3.63 | 40 | -50 | 61 | SPG(R) | -4.10 |  |
|  | 12 | -64 | 74 | SPG(R) | -3.59 | -45 | 49 | -6 | MFG | -3.72 |  |
|  |  |  |  |  |  | 28 | -94 | 17 | Omid(R) | -3.62 |  |
|  |  |  |  |  |  | -4 | 54 | -13 | LIFG | -3.61 |  |

**Supplementary Table 2.** Local maxima of brain processes (ERD/ERS) in the high compared with the low-lexical semantic control conditions (HC vs. LC, *p* ^FWE corr^*^.^* < 0.05) in the beta-band (from 0.1 sec. to 1 sec post-stimulus presentation). For each beta-band frequency or time window, the MNI coordinates (x, y, z) of local statistical maxima are given in mm, along with an approximate anatomical location and the corresponding t-value. Positive t-values indicate power increase (ERS) whereas negative values represent power decrease (ERD). A list of anatomical abbreviations is provided hereunder.

**List of abbreviations**

| **Location:** | |  |
| --- | --- | --- |
| **AG:** Angular gyrus  **CAL:** Calcarine  **CBL:** Cerebellum  **CBL1:** Cerebellum cru 1  **CBL2:** Cerebellum cru 2  **Cun:** Cuneus  **DLPFC:** Dorsolateral prefrontal cortex  **FUS:** Fusiform gyrus  **INS:** Insula  **ITG:** Inferior temporal gyrus  **IPL:** Inferior parietal gyrus  **LIFG:** Left inferior frontal gyrus  **LG:** Lingual gyrus  **MFG:** Middle frontal gyrus  **MTG:** Middle temporal gyrus | **O:** Occipital  **Osup:** Occipital superior  **PCL:** Paracentral lobule  **PostC:** Postcentral  **PrC:** Precentral gyrus  **rIFG:** Right inferior frontal gyrus  **SFG:** Superior frontal gyrus  **SFGmid:** Superior middle frontal gyrus  **SMA:** Supplementary motor area  **SMG:** Supramarginal gyrus  **SPG:** Superior parietal gyrus  **STG:** Superior temporal gyrus  **TH:** Thalamus  **PreCun:** Precuneus  **Omid:** Occipital middle gyrus | |
| **Side:**  All local maxima are located on the left side unless specified otherwise:  **(R): right-sided** | |  |


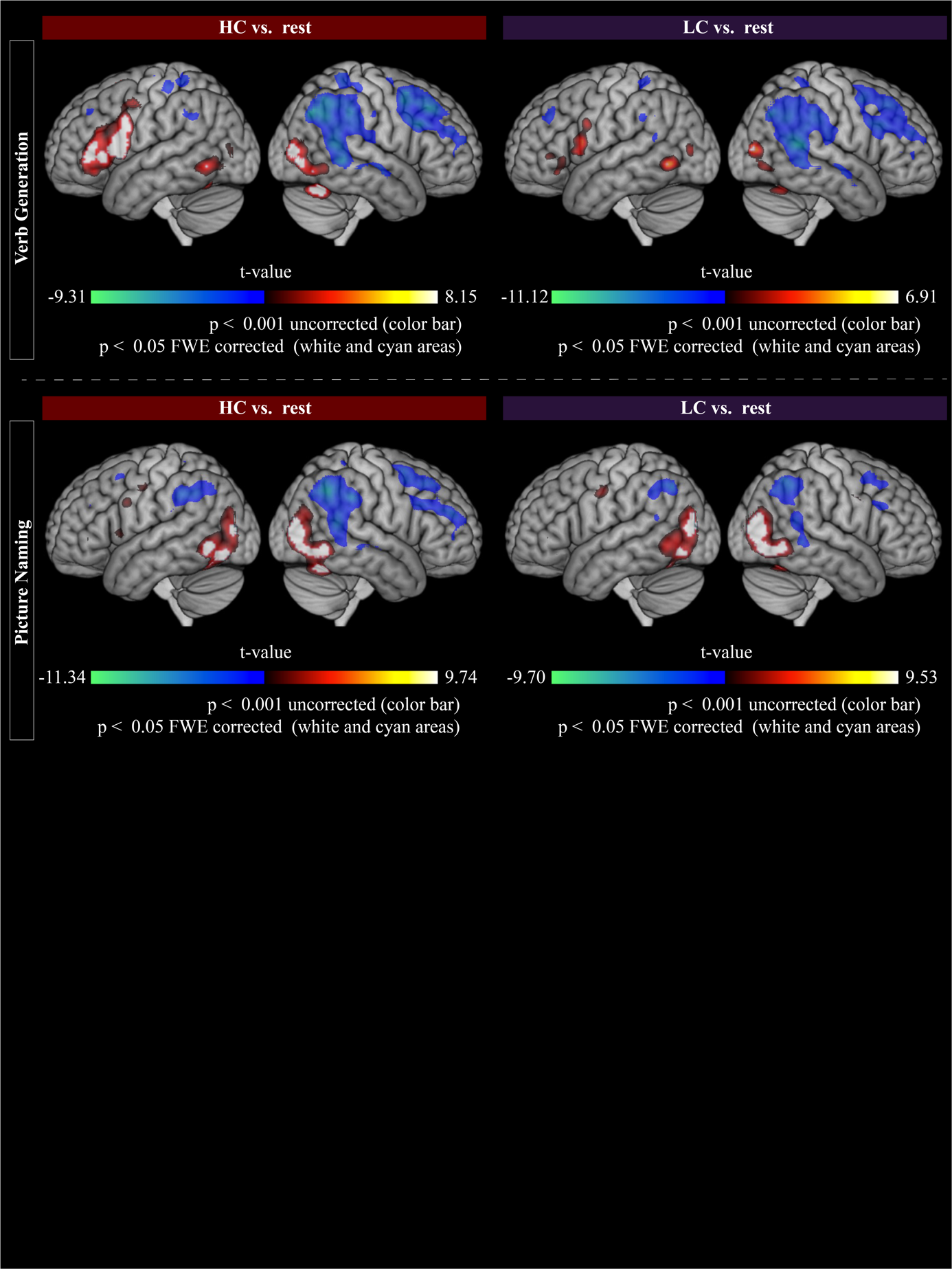


**Supplementary Fig. S1.** Whole-brain group-level statistical parametric maps for the VGEN and PN task. Mean fMRI activation maps showing increase (positive response; red) or decrease (negative response; blue) of blood level oxygen dependent (BOLD) signal in each lexical-semantic control condition (i.e., HC vs. rest and LC vs. rest). The color scale corresponds to voxel t-values and the map is masked statistically at *p* ^uncorr.^ < 0.001. Voxels surviving correction for the familywise error (FWE) rate using random field theory (*p* ^FWE^ ^corr.^ < 0.05) are shown in white and cyan overlays.

**Supplementary Fig. S2.** Brain maps displaying, for the verb generation (VGEN) trials, source locations showing significantly more pronounced power modulation of brain processes in the high lexical-semantic control condition compared with baseline (HC vs. baseline, *p* ^FWE corr^*^.^* < 0.05) in the beta-band (A. low beta; B. high beta) from 0.1 sec. to 1 sec post-stimulus presentation. For HC compared to baseline, relative more pronounced desynchronizations (ERD) in low or high beta are coded in a blue to green scale, while relative more pronounced synchronizations (ERS) are coded in a dark red to yellow scale.

**Supplementary Fig. S3.** Brain maps displaying, for the verb generation (VGEN) trials, source locations showing significantly more pronounced power modulation of brain processes in the low lexical-semantic control condition compared with baseline (LC vs. baseline, *p* ^FWE corr.^ < 0.05) in the beta-band (A. low beta; B. high beta) from 0.1 sec. to 1 sec post-stimulus presentation. For LC compared to baseline, relative more pronounced desynchronizations (ERD) in low or high beta are coded in a blue to green scale, while relative more pronounced synchronizations (ERS) are coded in a dark red to yellow scale.


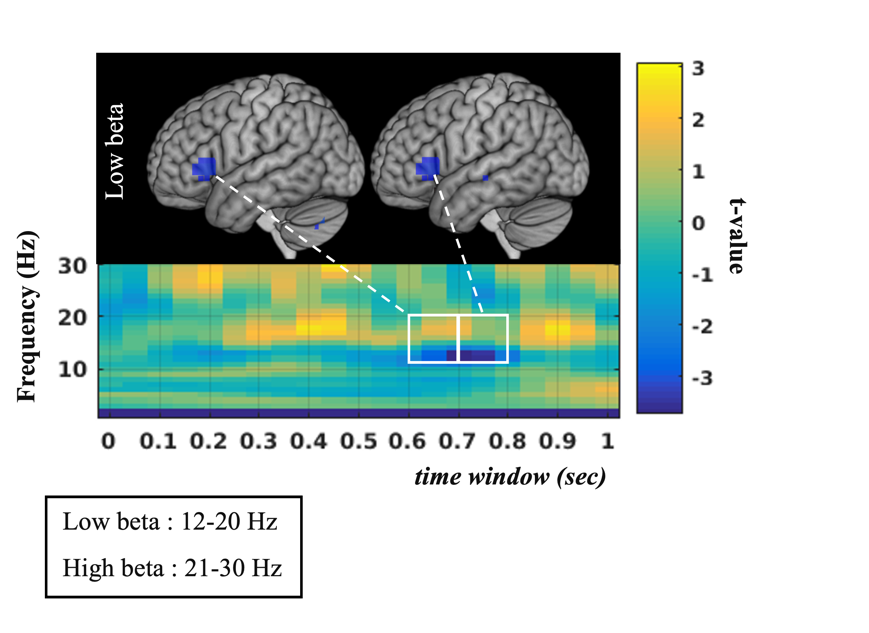


**Supplementary Fig. S4.** In the upper panel, brain maps displaying, for the verb generation (VGEN) trials, source locations showing significantly more pronounced power modulation of brain processes in the high compared with the low lexical-semantic control condition (HC vs. LC, *p* ^FWE corr.^ < 0.05) in the low beta-band from 0.1 sec. to 1 sec post-stimulus presentation. For HC compared with the LC condition, relative more pronounced desynchronizations (ERD) in low beta-band are coded in a blue to green scale, while relative more pronounced synchronizations (ERS and/or less pronounced ERD) are coded in a dark red to yellow scale. The lower panel shows the time-frequency representation (TFR) for Broca’s area, indicating significantly more pronounced low beta-band desynchronizations (ERD) between 0.6 and 0.8 seconds.
